# Psychosocial impacts of spontaneous coronary artery dissection: A qualitative study

**DOI:** 10.1371/journal.pone.0273978

**Published:** 2022-09-06

**Authors:** Barbara M. Murphy, Michelle C. Rogerson, Stephanie Hesselson, Siiri E. Iismaa, Robert M. Graham, Alun C. Jackson

**Affiliations:** 1 Australian Centre for Heart Health, Melbourne, Victoria, Australia; 2 Faculty of Health, Deakin University, Geelong, Victoria, Australia; 3 Melbourne School of Psychological Sciences, University of Melbourne, Melbourne, Victoria, Australia; 4 Victor Chang Cardiac Research Institute, Sydney, New South Wales, Australia; 5 St Vincent’s Clinical School, University of New South Wales, Sydney, Australia; 6 Center on Behavioral Health, University of Hong Kong, Pokfulam, Hong Kong; La Trobe University - Melbourne Campus: La Trobe University, AUSTRALIA

## Abstract

Spontaneous coronary artery dissection (SCAD) is an increasingly recognised cause of acute myocardial infarction, particularly in younger women without classic cardiac risk factors. While recent quantitative studies have noted high anxiety and depression in SCAD survivors, the full range and extent of psychosocial impacts of SCAD is unknown. The present study used a qualitative approach to investigate the psychosocial impacts of SCAD in Australian SCAD survivors. Focus group participants were recruited as part of a larger study of SCAD survivors currently being undertaken by the Victor Chang Cardiac Research Institute. Thirty SCAD survivors participated in one of seven online focus groups, conducted using a semi-structured format. Focus group duration was 1.5 hours. Each was digitally recorded and transcribed. Data were analyzed thematically according to recommended guidelines. One over-arching theme, five main themes and 26 sub-themes were identified. The over-arching theme related to lack of information, while the five main themes related to emotional impacts, issues with self-management, issues with family, impacts on work life, and the need for psychosocial support. The ‘emotional impacts’ theme comprised 11 sub-themes, namely shock and disbelief, confusion and uncertainty, unfairness, fear and anxiety, loss and grief, isolation and loneliness, guilt, invalidation and embarrassment, depression, vulnerability, and frustration. Findings are discussed in light of relevant psychological theories. This qualitative study extends previous quantitative investigations of SCAD survivors by providing an in-depth understanding of the complex, inter-related and highly distressing impacts of SCAD. The findings point to the urgent need for a coherent approach to information provision, the development and delivery of SCAD-specific cardiac rehabilitation programs, and the provision of psychosocial support programs for SCAD survivors.

## Introduction

Spontaneous coronary artery dissection (SCAD) is an increasingly recognised cause of acute coronary syndrome (ASC) and sudden cardiac death in people without classic cardiac risk factors [1, 2]. Typical acute myocardial infarction (AMI) is caused by thickening or hardening of the arteries due to plaque buildup, known as atherosclerosis, that can abruptly break away from the arterial wall. This results in the rapid formation of a blood clot, thereby obstructing blood flow. SCAD, however, is non-atherosclerotic and occurs when a coronary vessel develops a hematoma within the arterial wall which bulges inwards, obstructing blood flow [3]. SCAD is associated with underlying systemic vascular conditions such as fibromuscular dysplasia (FMD), connective tissue disorders, migraine headache, and inflammatory disorders [3–8]. Emotional stress and physical exertion are often precipitating factors to SCAD events [4–6, 8–11]. SCAD is also associated with peripartum status [8, 12].

SCAD predominantly affects younger women and accounts for a large proportion of AMIs in this group [2, 3, 5]. While the prevalence of SCAD remains uncertain due to underdiagnosis, recent estimates suggest that SCAD accounts for up to 4% of ACS cases overall and up to 35% of AMIs in women ≤50 [3, 13], and is the most common cause of pregnancy-related AMI [8]. It has recently been noted that ‘SCAD is far more common than previously thought’ [3] and is ‘no longer so rare such that it cannot be studied’ [13].

Emerging evidence suggests that SCAD is a particularly stressful event, with high rates of anxiety and depression [10, 14–17], stress, burnout and fatigue [11, 17], and post-traumatic stress [15, 17] reported in quantitative studies. There is some evidence that SCAD survivors have higher levels of anxiety, depression and distress than those who have typical AMI due to atherosclerotic coronary artery disease [16], although a recent study did not support this [18].

It has been postulated that SCAD is a particularly stressful event due to its rarity, sudden onset, unclear pathogenesis, uncertain optimal acute and long-term management, and likelihood of recurrence [3, 19]. In the absence of atherosclerosis and traditional risk factors for cardiovascular disease, SCAD comes as a shock to survivors and is not suited to traditional lifestyle management approaches typically seen in cardiac rehabilitation (CR) settings [20, 21]. Evidence also suggests that the information provided to SCAD survivors is ‘insufficient or inadequate’ [10], with a reliance on the Internet for information [10].

To date there have been few attempts to understand the specific experiences of SCAD survivors and the reasons why SCAD is so stressful. The one exception is a Canadian study that used qualitative methods to explore the challenges faced by SCAD survivors, highlighting anxiety, uncertainty, isolation, identity challenges, and family stress as common challenges [22]. However, more qualitative research is needed to delve more deeply into the full range of psychosocial experiences of SCAD survivors. Recent reviews have noted that SCAD remains relatively under-researched and poorly understood, and requires greater research attention [3, 13].

The present study used a qualitative approach to investigate the experiences of SCAD survivors, with a focus on the psychosocial impacts of SCAD. The aims of the study were to provide an in-depth understanding of the nature of the stresses and concerns faced by SCAD survivors, and to identify their support needs and preferences.

## Method

### Recruitment

Participants were recruited by the Victor Chang Cardiac Research Institute from a data base of participants in a larger genetic study (referred to here as the VC Genetics Study). All participants provided written consent to participate in the focus group study as part of the consent process for the VC Genetics Study. Eligible participants had had an episode of SCAD in the preceding 12 months. A list of eligible participants was compiled by the VC Genetics Study Co-ordinator (SH), who then emailed or telephoned participants to seek their involvement in the current study. Of 50 people contacted, 32 agreed to participate. Participants were presented with a selection of five dates with both daytime and evening options. Two potential participants were unavailable on the given dates. The study was approved by the St Vincent’s Hospital Sydney Human Research Ethics Committee (HREC/16/SVH/338).

### Procedure

A total of 30 SCAD survivors participated in one of seven focus groups, undertaken using the Zoom videoconferencing platform, with group size ranging from three to six participants. Discussions were facilitated by two investigators (BM and MR) with extensive experience in focus group facilitation. Discussions followed a semi-structured format to address: precipitating factors; impacts of SCAD on physical health, psychological wellbeing, family life, work life, physical activity levels, and pregnancy decisions; issues regarding CR, resuming physical activity and following lifestyle recommendations; issues regarding prognosis and recurrences; and support needs and preferences. Focus group duration was 1.5 hours. With consent of all participants, each discussion was digitally recorded and transcribed in full. Participants were not offered a financial incentive.

### Analysis

Three investigators (BM, MR, AJ) independently read the transcripts to identify broad themes and sub-themes related to psychosocial impacts of SCAD. These were then discussed to achieve consensus. Thematic analysis was undertaken following recommended steps [23, 24]. Investigators increased familiarisation with the data via audio playbacks and repeatedly moved between reading, reflecting, and interpreting the transcripts. Data were coded into themes and sub-themes. Investigators then discussed and compared codes, reflected, and refined the themes. The analysts all had strong knowledge of CR, and extensive experience in conducting qualitative research, ensuring reflexivity and rigour. Themes are presented with verbatim illustrative quotes from participants.

## Results

### Participant characteristics

Participants ranged in age from 35 to 71 years, with mean (SD) age of 52.2 (9.5) years, median of 52.5 years. Other participant characteristics are shown in Table 1.

**Table 1:** Participant characteristics.

Characteristic (N = 30)	*n*	%
*Sex*		
Female	27	90
Male	3	10
*Partner status*		
Partnered	27	90
Unpartnered	3	10
*Employment status*		
Employed	27	90
Not in paid workforce	3	10
*Living arrangements*		
Live with others	29	97
Live alone	1	3
*Time since SCAD event*		
1–2 months	3	10
3–4 months	5	16
5–6 months	6	20
7–8 months	6	20
9–10 months	2	7
11–12 months	8	27

*N* = 30. Note SCAD = Spontaneous Coronary Artery Dissection

As shown in Table 1, participants were most often female (90%) and married or partnered (90%). Most (90%) were employed. Almost all participants lived with others (97%), the majority living with their partner and children (62%) or their partner only (28%). Participants resided across most states of Australia, with the majority living in New South Wales (43%) and Victoria (30%). All participants had experienced their SCAD in the previous 12 months, with the average time of approximately 7 months (mean = 6.9 months, SD = 3.4, median = 7.0).

### Themes and sub-themes

#### Overarching theme—lack of information

The major and overarching theme that emerged was lack of information about SCAD, including information related to its cause, management and prognosis. Participants talked extensively about lack of knowledge about SCAD amongst their treating health professionals, from the time of presentation at the hospital emergency department through to later consultations with cardiologists and general practitioners (GPs). Participants stated that many health professionals *‘had never heard of SCAD’* and often *‘knew nothing about it’*, and commented that health professionals were not aware of differences between SCAD-related heart attack and typical atherosclerotic heart attack:

*“the health professionals have no idea what it is*. *No-one knows anything about it. They say ‘oh it’s a heart attack, you have to change your diet’. But it’s not that.”*

Given the lack of knowledge amongst medical professionals, participants had received very little information about SCAD. Some had received *‘absolutely zero information’*, with many commenting that *‘nobody told me anything’*. Participants talked about receiving conflicting information from different health professionals: *“I had two specialists tell me two different things*. *That was a bit of a struggle”*. For many, the ‘knowledge deficits’ and ‘information scarcity’ was the worse aspect of their experience:

*“the worst thing is the lack of credible*, *solid information, consensus about what causes it, what’s the best way to deal with it, how do we manage it moving forward”*

Very few participants had received any written information about SCAD. Instead, most had received general information about typical atherosclerotic heart attacks: *“the GP just puts you in the heart attack basket”*. This left people floundering for information:

*“the lack of information*, *it’s exasperating, it’s frustrating, it’s tiresome. And it means that we’ve basically had to do all our own research on this. But you don’t know where to look, and you don’t know what you’re looking for”*

**Fig 1:**
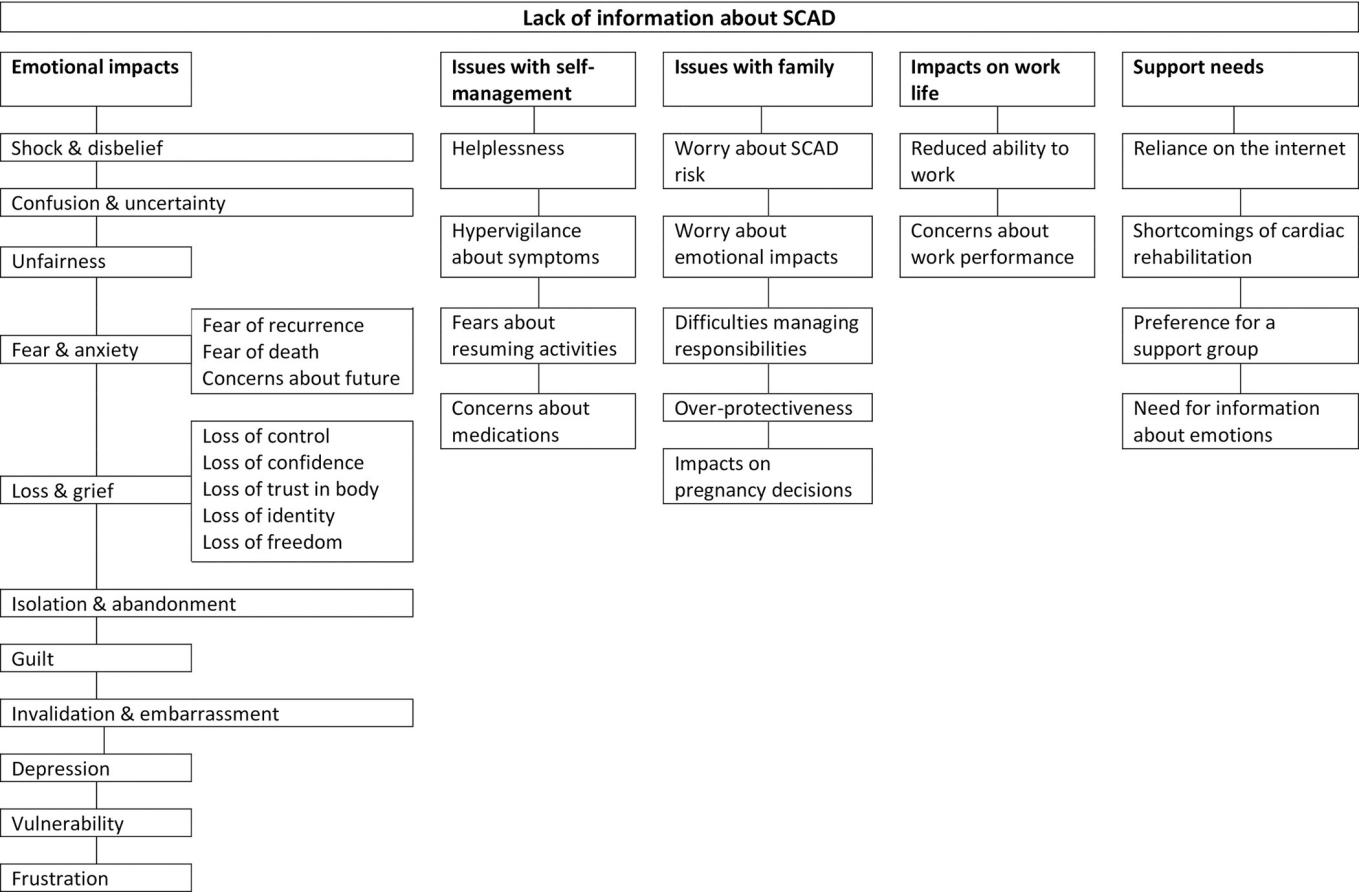
Themes and sub-themes.

This issue dominated discussions and impacted on all other themes and sub-themes that arose. The over-arching theme, together with the major themes and sub-themes, are shown in Fig 1.

#### Major theme 1. Emotional impacts

Participants reported a range of emotional impacts of SCAD. Broadly these included confusion at the time of the event and beyond, shock and disbelief once diagnosed, followed by uncertainty related to limited or conflicting information in terms of management and prognosis. Other emotions included fear and anxiety, loss, isolation and abandonment, depression, guilt, invalidation and embarrassment, a sense of unfairness and vulnerability. Participants referred to the *‘roller-coaster of emotions’*, with an array of intense and fluctuating emotions over the recovery period.

*1a*. *Shock and disbelief*. Once diagnosed with SCAD, participants were commonly in a state of shock and disbelief because the SCAD event was sudden and unexpected. Because of its non-atherosclerotic nature, the event occurred to people as *‘random’*, *‘spontaneous’* and *‘something just out of the blue’*. Most had not seen themselves as a candidate for heart attack, typically describing themselves as *‘not at risk’* because they were *‘fit and healthy’*. Some had previously been reassured by their GP that they had very low heart disease risk because they had no risk factors—*‘he said I was in excellent health’*—which exacerbated feelings of shock when faced with the SCAD diagnosis.

*1b*. *Confusion and uncertainty*. For many participants there was considerable confusion at the time of the event, due to their lack of familiarity with SCAD, relatively young age, and absence of traditional CVD risk factors. Many commented that they *“didn’t know that there are other types of heart attacks”* and that they therefore *“didn’t think it could be a heart attack”*. Participants tended to interpret their symptoms as non-cardiac, attributing them to heartburn, “gastro”, strained muscles, slipped disc, appendicitis, or anxiety. On presentation at hospital, the symptoms were also often regarded by health professionals as non-cardiac, which created delays in diagnosis and treatment. Participants sometimes stated that they *“felt like a fraud”* due to the confusion at the time of diagnosis.

Participants remained confused well into their recovery period due to a lack of clarity about SCAD, many stating that *“no-one seems to know what it is or how to manage it”*. There was confusion about the underlying causes of SCAD, including about the role of fibromuscular dysplasia (FMD), hormone issues, migraine and other conditions. Similarly, there was confusion about the potential triggers for the acute SCAD event. Participants commonly stated that lack of consensus about causes and triggers was *“frustrating”*. The apparent rarity of SCAD added to confusion about the diagnosis, leaving people with an unanswered question about *“why me*?*”*. This was particularly the case for male participants who commented that they were an *“outlier”* or *“out of the norm”*. Confusion about management and prognosis, including recommendations regarding resuming daily activities and exercise, and potential for recurrence, was extremely common.

Participants highlighted the *“the uncertainty*, *the not knowing”* as being amongst the worst aspects of having had SCAD. Uncertainty was tied specifically to having received inadequate or conflicting advice from health professionals.

*“there’s a lot of uncertainty, in what should happen, what shouldn’t happen, what is or isn’t the treatment. There seems to be no continuity. Whether that’s due to lack of information, lack of knowledge from cardiologists. My GP didn’t even know what a SCAD was”*.

Some participants even stated that they would have preferred to have a more typical type of heart attack to avoid the uncertainty and the unknown trajectory of SCAD:

*“I wish I had had a traditional heart attack*, *I wish I had had a stent or stitches up my chest or something, because then I would be fixed”*.

*1c*. *Unfairness*. The lack of risk factors for a traditional heart attack also created a sense of unfairness for some people, with people commenting that *“I’d been looking after myself*, *so ‘why me*?*’*”. Some compared themselves to people who had numerous ‘traditional’ risk factors, commenting *“it isn’t fair that it’s happened to me”*. This created feelings of annoyance and anger because they *“did everything right”*.

*1d*. *Fear and anxiety*. Almost all participants talked about a general sense of anxiety and fear in the aftermath of SCAD. While for some fear and anxiety had abated over time, for others the anxiety had been chronic and persistent. Lack of knowledge of SCAD amongst health professionals exacerbated anxiety: *“when the people who are looking after you don’t even know what they’re doing*, *it’s very scary”* and “*It adds to the anxiety because you don’t feel that you are getting the best care*, *or the best advice”*. Fears related specifically to recurrence, the future and death, forming three sub-themes:

**1d(i) Fear of recurrence**
Fear of having another SCAD event was very top-of-mind for many participants and was “*constantly hanging over your head”*, *“an over-riding fear”*, *“all-consuming”* and, for some, *“the worst thing about it”*. The concerning statistics relating to recurrence exacerbated this fear: *“when you read that you’ve got a huge chance of dropping dead*, *that’s scary”*. People talked about fears of having another event at work, whilst driving, whilst at home alone, whilst with their children, and whilst travelling. Fear of recurrence was exacerbated by lack of knowledge about SCAD’s causes and triggers: *“You don’t know when it will happen again”*. Many people avoided activities, particularly those involving lifting or requiring physical exertion, for fear of triggering another SCAD. Participants raised concerns about travelling far from home or being a long distance from hospital: *“I’m hesitant to go on a long trip*, *even a long drive*. *I feel I’ve got to stay close to where I can get to hospital quickly if I need to”*. Some people had become hypervigilant about *“constantly checking my blood pressure and heart rate”* while doing activities, again due to fear of triggering an event. Some commented that they were *“scared of getting upset or agitated”* and *“stopped (myself) from crying or getting angry”*, explaining that they avoided stressful situations or emotional arousal for fear of triggering another SCAD. Some were considering taking anti-anxiety medication to reduce the likelihood of anxiety triggering another SCAD event. Many people talked about having readmitted themselves to hospital thinking they were having another SCAD.
**1d(ii) Fear of death**
Participants talked about their heightened fear of death since having SCAD, commenting that they were *“more worried about death”* and *“more conscious of dying”*. Most stated that they *“didn’t think about death until the SCAD”*. Some raised the issue of death in terms of a realization that they had narrowly escaped death.
**1d(iii) Concerns about the future**
In addition to specific fears of recurrence and death, participants mentioned a more general fear about the future commenting that they felt *“uncertain and dubious about what the future held*, *and how are you going to recover from this*?*”* and *“having to live with this concern*, *this worry*, *hanging over my head*. *That makes me anxious about the future”*.

*1e*. *Loss and grief*. Participants described many losses they had faced since having SCAD, which were grouped into four sub-themes. Losses related to control, self-confidence, trust in the body, and identify, forming four sub-themes:

**1e(i) Loss of control**
Participants commented that they had had to relinquish control over certain activities or responsibilities, including physical activities, household duties, and other choices and decisions: *“loss of control of the most simple things*, *like where you go*, *what you do”*.
**1e(ii) Loss of self-confidence**
Participants talked about effects on their self-esteem, commenting that they had *“lost confidence in day-to-day living”*. For some participants, weight gain associated with medications and, to a lesser extent, a reduction in physical activity had impacted on self-esteem. Participants often commented that weight gain was *“definitely taking its toll on me”*, noting that “*it’s very depressing”*, *“it’s embarrassing”*, *and “I hate it*, *it’s terrible for my self-esteem*.*”*
**1e(iii) Loss of trust in the body**
Participants sometimes commented *“my body has let me down”* and, again, this was exacerbated by having SCAD in the context of being fit and healthy: *“the hardest thing is trusting my body again*. *I just relied on it so heavily throughout my entire life”*.
**1e(iv) Loss of identity**
Participants talked about the negative impacts of SCAD on self-perception and identity and having to come to terms with and accept that they are *“not the same person as before”*. Again, this was particularly challenging for those whose identity had been tied up in physical abilities, including those who had been elite sportspeople: “*it’s such a huge blow to your image of yourself*, *and your image of how your life is going to be*. *It’s been hard”*. It was also heightened for those who had been high achievers in their professional life: *“My whole sense of identity is about working hard*, *pushing myself*, *achieving things*. *And this has taken a lot of that away*, *because I just can’t do that anymore*. *It’s had a big impact”*. Some indicated that SCAD defined their life: *“It’s like a fork in the road*. *Everything in my mind now is ‘before’ and ‘after’ that episode”*.
**1e(v) Loss of freedom**
Many participants talked about restrictions on their lifestyle due to SCAD, highlighting this as amongst the worst and *“most confronting”* aspects of the event: *“I could always do what I wanted to do and not having that carefree attitude or approach*, *I find that the hardest part*, *mentally”*. These restrictions ranged from more strenuous activities such as “*not being able to do all the exercise I want to do”* or *“not being able to get back to playing soccer”* to more mundane things such as “*being told I can’t even vacuum”* or “*all of a sudden you can’t even mow the lawn”*. There was often a sense of grief associated with not being able to exercise at their pre-SCAD levels: *“How do I manage that*? *Being told that there are certain things that I’ll just never do again”*. For some, this was life-changing:

*“the switch has been flipped and the life that I had before no longer exists. It’s a new life. It’s transitioning and adjusting to that. And at (young age), that’s a lot to take on”*.

*1f*. *Isolation and abandonment*. Lack of information and support in hospital and after discharge led to feelings of isolation, with participants commenting: *“you go home and you’re on your own*. *You’re basically navigating it alone”* and *“it’s pretty isolating”*. Many participants had little or no emotional support during their recovery, which exacerbated feelings of isolation, and commented that they felt *“completely abandoned”* by health professionals. One expressed this strongly:

*“I felt completely deserted once I left hospital, completely. I was very scared and looking for someone to give me some guidance, and there was nobody. I felt completely deserted. From the hospital and the doctors, no-one knew, no-one was telling me anything”*.

*1g*. *Guilt*. Participants talked of feelings of guilt, which again was often exacerbated by lack of knowledge of the cause of SCAD or a feeling that they had caused it themselves: *I thought “what have I done wrong*?*”* and *“I was stressed at work*, *I was working too hard*. *There is guilt for that”*. Some also talked of feelings of guilt *“because I’d put my family through so much distress”*.

*1h*. *Invalidation and embarrassment*. Many participants explained that, because of the non-atherosclerotic nature of SCAD, other people–including family, friends and health professionals—often minimised the event and commented that *“it wasn’t a proper heart attack”*. Common misconceptions about SCAD prompted others to assume that the person had traditional risk factors, which left people feeling invalidated, misunderstood, and dismissed:

*“People go ‘oh*, *but it wasn’t really a heart attack’. That really gets my back up. Because it was a heart attack, it was just caused by a different mechanism”*

*“They say ‘well, you’re probably a bit overweight, do you have cholesterol, what’s your blood pressure like?’ Well actually everything is fine, this has nothing to do with your traditional plaque in your arteries*. *But you have to explain that. It makes me annoyed”*

Participants sometimes indicated feeling embarrassed for having a heart attack as it implied that they were making unhealthy decisions or not looking after themselves, which left people *“at great pains to explain that it’s not a normal heart attack*, *I don’t have atherosclerosis and I’m actually healthy”*.

*1i*. *Depression*. While few participants talked specifically of having experienced clinical levels of depression, many talked about low mood, feeling *“pretty sad”* and being *“on the verge of depression”*. Some mentioned factors indicative of depression, such as losing pleasure in activities they had previously enjoyed. Several talked about fluctuating mood, commenting that *“your moods are all over the place” and* “there are good days and bad day”:

*“you are all over the shop*, *emotionally and mentally. You do get a bit teary, because it’s such an overwhelming thing to be hit with. It’s hard to stay positive”*

*1j*. *Vulnerability*. Participants talked about a heightened sense of vulnerability since SCAD, noting that *“you have to suddenly think of yourself as a vulnerable person”*. Some noted a more acute awareness of the fragility of life: *“There is a sense that life is pretty fragile*. *A sense of vulnerability*. *That unknown again*. *That stuff you can’t control*. *We don’t know what’s going on in our bodies”*.

*1k*. *Frustration*. Almost all participants commented about extreme fatigue and breathlessness in the weeks and months after SCAD, with many commenting that they were *“completely washed out”* and *“very*, *very tired and breathless”* with some complaining that they were *“knackered all the time*!*”*. This was particularly challenging for people who had, on the whole, been fit and physically active prior to SCAD, with many noting that they felt *“completely frustrated”* about their level of fatigue. Some noted that “*fatigue has been the biggest thing*, *not being able to do the things I would normally do”*.

#### Major theme 2: Issues with self-management

Participants described a range of difficulties regarding self-management of their condition, again often linked to inadequate or conflicting information.

*2a*. *Helplessness and incompetence*. Due to its non-atherosclerotic nature and in the absence of traditional CVD risk factors, SCAD left people feeling helpless in managing their condition: They noted that SCAD-related heart attack is *“harder to manage because you can’t blame it on things you can change”* and *“there’s no way you can fix it by the normal heart attack strategies”*. This lack of control created fear:

*“normally with a heart attack, you get back to exercise, eat right, and you can fix this. But with SCAD I thought ‘how am I going to fix this? How am I going to help myself?’ I didn’t know, so that’s why I was frightened”*.

*2b*. *Hypervigilance about physical symptoms*. Participants indicated that since their SCAD they had become highly focussed on their body and physical symptoms, including racing pulse, irregular heartbeat, and chest pain. Many had experienced *“shocking and persistent chest pain”* since the SCAD, noting that this was *“very disconcerting”*. Many had become *“hyper-alert and hyper-sensitive to anything that happened in my body”*. They commented that this hypervigilance was *“tiring”* and *“made me feel like I was probably seen as a hypochondriac”*. Side-effects of medications sometimes mimicked signs or symptoms of heart attack, exacerbating fear and concern: *“all that sensation around your chest*, *and heartburn*, *it just freaks you out”*. Some participants had decided to stop taking certain medications due to the intensity of side effects.

*2c*. *Fears and confusion resuming usual activities and exercise*. The issue of when and how to resume usual activities and exercise was a great concern for participants, with a lot of confusion and fear related to doing any type of physical or strenuous activity. Many commented that they avoided activities, often asking themselves *“is this the thing that is going to kill me*?*”*. Fears related to extreme levels of exercise such as hiking and bike-riding, as well as more minor activities such as lifting children, doing housework or gardening, and doing moderate activities such as walking or jogging. Many commented that they had previously been *“very independent”* but now *“have to rely on other people to do the most basic things”*, which often caused frustration *“because you can’t do what you used to do”*. The constant internal questioning around whether or not to resume certain activities, or to undertake certain tasks, was particularly frustrating for some people.

*“It’s that constant analysis about whether something you want to do is something that you can or should do*. *Whereas before you’d just go and do it, without a second thought. It is a big adjustment””*

Again participants often compared themselves to non-SCAD heart attack counterparts.

*“my friends who had (a traditional heart attack)*, *they have healed and are living their life, they’re running, they’re doing all the things they used to do. Whereas I’m still a bit timid about getting back to that kind of physicality in my life. So that’s what I’ve found the hardest”*

*2d*. *Concerns about medications*. Several participants were overwhelmed and upset about the number of medications they had to take, with many commenting that they had never taken medications prior to SCAD and that it was *“a big adjustment”* and “*really upsetting”*. Many expressed concerns and confusion about their medication regime due to their perception that that these traditional CVD risk-lowering medications were not relevant for them: *“why am I on cholesterol medication when I don’t have a cholesterol problem*?*”*. Some were concerned that prescribed blood thinning medications were contraindicated: “*you don’t want a drug like aspirin that’s going to encourage bleeding”*.

Lack of knowledge about SCAD amongst primary care physicians exacerbated medication concerns: *“nobody has any idea*, *they push you to the side and go ‘Here*, *take this medication*, *you’ll be fine’”* and *“they put you on the quintuplet thing of the usual bypass meds"*. Some people had made their own decisions to stop taking certain “unnecessary” medications:

*“I decided to come off the medication*. *You feel a little bit like you’re doing your own research and making your own decisions. That’s what I’ve been doing anyway”*

*“I said to my GP ‘look*, *I don’t need to be on these statins, my cholesterol is fine, this is not an atherosclerotic event. I’m not taking them’. And he said: ‘I can understand, yeah stop’. So I was off them within a week”*

Many participants talked about physical symptoms which they attributed to side effects of medications. These commonly included breathlessness, fatigue, weight gain, memory problems and “brain fog”, which were often attributed to medications for blood pressure and cholesterol, including beta-blockers and statins. Less common symptoms included indigestion, neck pain, difficulty swallowing, nightmares and mood swings. A handful of women also highlighted difficulty in managing menstruation due to blood thinning medication, stating *“periods are impossible to manage”*.

#### Major theme 3: Family impacts

Several participants were dealing with concerns related to family members, including their potential risk of SCAD, emotional impacts, difficulties managing family responsibilities, and over-protectiveness of family members.

*3a*. *Concerns about SCAD risk in family members*. In light of known genetic links in SCAD, participants raised concerns about their children’s risk of SCAD, with concerns for their children often outweighing their own personal fears: *“My biggest worry is not my life*, *it’s theirs*, *it’s my kids*.*”* Concerns for their adult daughters and pregnancy were raised, given that SCAD is associated with peripartum status. *“I’m very worried about my two daughters when they have children*, *and beyond*, *it’s a big concern”*.

*3b*. *Worry about emotional impacts on family*. Participants talked about worry and concern experienced by family members, who were *“stressed and anxious because I’d had a heart attack”*. These concerns were greatest for those with young children and were particularly acute for women who had been the major caregiver in the family: *“you’re the mum*, *you’re the one who does everything*, *you’re not meant to be sick”*. Concerns about the consequences for children who had witnessed the event were also raised: *“they were terrified and panicked”* and *“they’ve seen things that teenagers shouldn’t have to see”*. The mental health impacts of SCAD had put additional stress on family members: *“With the emotional roller coaster that I have*, *my husband feels like he’s walking on eggshells the whole time*. *He doesn’t know which (me) he’s going to get*. *It’s hard for him”*.

*3c*. *Difficulty managing family responsibilities*. Participants talked about the stress of multiple roles and difficulties managing family responsibilities in the aftermath of the SCAD:

*“All the internal pressures of life still continue: running a house*, *managing the kids, budgeting, that just continues. So often you think ‘I just need a break. I just need to recover. I just need some time, some space’. And you don’t get any of that. For me it’s been pretty bumpy”*

For some, SCAD had created tensions in the family related to household tasks and other changes, with some commenting the SCAD had put “extra burden” on other family members.

*3d*. *Over-protectiveness*. For some participants, family members, sometimes including young children, had become over-protective. Some talked about being *“wrapped in cotton wool”*, *“being monitored”*, and *“fussed over”*, and feeling *“they were treating me like I was fragile”*. Some stated that this was frustrating: *“It frustrates me sometimes*, *you do get a bit angry*, *you want your independence”*.

*3e*. *Decisions regarding future pregnancy*. For a small number of participants, SCAD had impacted on future decisions about pregnancy and having children: *“We were just starting to think about trying for a second child*, *and my cardiologist has said that’s out of the picture now*. *So that’s been a big emotional burden that we’ve had to come to terms with*.

#### Major theme 4: Impacts on work life

*4a*. *Reduced ability to work*. SCAD impacted negatively on work in a variety of ways. Some participants had missed new work opportunities, while others had reduced or modified work hours or tasks to reduce their work pressure since the SCAD. Some had retired or stopped paid work, noting either that their work capacity had decreased: *“I can’t do what I used to do*, *which is hard to accept”*, or that they had brought retirement forward: “*I used it as an excuse really”*. Others felt they had returned to work too early and were not ready, or would have preferred to work less but were not in a position to do so. Related to work impacts, participants also talked about the financial impacts of SCAD: *“there’s a financial impact*, *a massive one*. *The financial impact was just terrible”*

*4b*. *Concerns about work performance*. Participants worried that they were not performing as well at work compared to pre-SCAD, or were left questioning their ability: *“It definitely makes me question whether I can give as much as I used to*, *to my workplace”* and *“can I seriously give my job my best now*?*”*

#### Major theme 5: Information and supports

As noted, a lack of information was an over-arching theme that permeated all discussions and impacted on all other areas. Specific themes related to information and supports emerged, including a reliance on the internet, shortcomings of CR programs for SCAD patients, and preferred supports.

*5a*. *Reliance on the internet*. As they were rarely provided with information from health professionals, participants relied heavily on the internet for information during early recovery: *“if it wasn’t for Google*, *I wouldn’t have known anything*. *I would have been literally on my own”* and *“You become obsessed about searching on the internet; I was obsessed about reading everything I could about it”*

In their search for information online, participants often named the Victor Chang Cardiac Research Institute website and the MAYO Clinic website as their preferred sources of credible information. The Australian SCAD Research Inc. Facebook group was also highlighted as being helpful, particularly for inspiration and information: *“It’s a wealth of information”*, and for feeling that you are not alone in having had SCAD: *“to hear from people who have had similar experiences”* and “*it’s that bit of encouragement*, *that there is light at the end of the tunnel”*. While social media, including Facebook, was useful for obtaining information, most participants did not gain emotional support through this medium: *“you gather information*, *but emotional support*, *no”*.

*5b*. *Shortcomings of cardiac rehabilitation programs*. Most participants had either chosen not to attend CR as they did not think it would be relevant for them, had been advised by a health professional not to attend as it *“wasn’t suitable”*, or it was not made available to them. A small number of participants had attended CR and most had found it to be irrelevant to their needs: *“it’s very annoying because lots of things they’re saying and advising you*, *it’s not relevant for you”*. Limited knowledge amongst CR staff undermined participants’ confidence: *“You lose all faith in them and in everything they tell you”*. Participants commented on the older age and poorer health of the other CR attendees, which highlighted their feelings of lack of belonging and isolation, and left them feeling like *“a fish out of water”*. On the whole participants noted that traditional CR *“doesn’t cater for SCAD”* and *“the information you receive is not relevant*.*”* Many commented that they needed SCAD-specific CR: *“I would like to see some sort of rehab program for SCAD patients”*. Only a handful of participants who had attended CR had found a few aspects of it to be helpful.

*5c*. *Preferences for support group*. When asked about support preferences, participants stated that being with other SCAD survivors would be beneficial, both for information exchange and emotional support: *“being able to talk about it with people who are going through the same thing*, *exchanging information*, *share your fears and worries*, *that would be very*, *very useful”*. They noted that it would be beneficial to have contact with people who have recovered and resumed their usual activities and exercise, for inspiration and support: *“that light at the end of the tunnel*, *that it’s not all doom and gloom”* and *“having others who have gone through it is by far one of the greatest supports*, *because they have lived it*. *The more of the stories you can hear*, *the more you get the big picture”*. Participants specifically noted that a support group would be helpful, particularly with facilitation by a health professional and/or SCAD survivor.

*5d*. *The need for information and support in hospital*. Participants commented of the need for more emotional support in hospital, including being warned about the stressfulness of the recovery period: *“The emotional side of it*, *I think it needed to be talked about a bit more in hospital*, *before going home”*. Participants also suggested that information about likely emotional impacts of SCAD should be provided on discharge from hospital, together with referral for emotional support with counsellors specialising in SCAD:

*“You need to be given something as you leave that tells you that you are possibly going to suffer anxiety*, *you’re going to have an emotional rollercoaster because of this. There’s not enough information as you leave hospital”*.

Other suggestions included: *“a brochure with phone numbers of counsellors you can talk to who are specialists in SCAD”* and *“an organisation that specialises in the emotional impacts of SCAD*, *that would be empowering”*.

## Discussion

The present study has identified several major themes and sub-themes related to psychosocial impacts of a SCAD-related heart attack for survivors. The over-arching theme of *lack of information* impacts on all areas, highlighting the way in which lack of knowledge about and understanding of SCAD amongst health professionals results in insufficient provision of information and support to SCAD survivors, exacerbating the emotional impacts of SCAD. While lack of adequate information has been reported previously [10], our study goes further by demonstrating its impacts on SCAD survivors’ emotional wellbeing and overall recovery.

Amongst the most challenging emotional impacts was uncertainty about SCAD, including diagnosis, treatment options, return to daily living and physical activity, prognosis, and risks of another event. This finding reinforces that of the only other qualitative study in this field, which similarly identified navigating uncertainty as a major challenge for SCAD survivors [22]. The importance of uncertainty in illness has received considerable attention over the past three decades and has been investigated in both cardiac [25, 26] and non-cardiac [27] chronic conditions. Mischel’s *Model of Uncertainty in Illness* proposes that uncertainty results when the patient is unable to determine the meaning of the illness or diagnosis, or is unable to accurately predict the likely trajectory and outcomes [28]. Uncertainty is particularly high in illnesses with unclear diagnoses, treatments and management guidelines, and in those with high risk of recurrence [25, 27, 28]. It is not surprising, therefore, that uncertainty is high following SCAD given its relative rarity, unclear cause and trajectory, lack of consistent guidelines for management, and high recurrence rate [3, 13, 19]. Uncertainty is associated with lack of information provision, and leads to reduced capacity for self-management, poor quality of life, and increased risk of depression [25, 26, 29]. Such uncertainty was clearly evident in our focus group findings: participants were left feeling confused and helpless in their self-management efforts, with a sense of having little control over their risk of another event.

The broader array of emotions experienced by SCAD survivors in our study can be categorised and understood within Self-Determination Theory (SDT), which has gained increasing attention in relation to the self-management of chronic conditions [30, 31]. According to SDT, personal wellbeing and effective self-management involves the fulfilment of three basic psychological needs: autonomy, competence and relatedness. Autonomy involves having control over one’s behaviour and choices in life; competence involves having a sense of mastery and efficacy; and relatedness involves feeling accepted and understood [30, 31]. Our findings show that each of these three areas is threatened following SCAD. First, there is a threat to *autonomy*: participants felt a loss of control over their body and their health, and a loss of freedom to be the person they had previously been and to live as they had previously lived. That SCAD cannot be managed through lifestyle change exacerbates feelings of helplessness in being unable to ‘fix’ the condition or actively minimise the risk of recurrence. Second, a sense of *incompetence* arises: participants felt confused, anxious, fearful, vulnerable, frustrated, depressed and, as already noted, extremely uncertain. Consequently, there is an eroding of previously intact self-confidence and self-esteem, resulting in a lack of mastery and efficacy to manage the condition and other aspects of life. Third, there is a lack of *relatedness*: participants felt unsupported, invalidated, misunderstood and isolated, largely due to a lack of understanding of SCAD amongst health professionals and within the general community.

Our findings also highlight loss of one’s previous lifestyle, and grief associated with that loss, as a major impact of SCAD. Reconciling pre- and post-SCAD identities has been identified previously as a major challenge faced by SCAD survivors [22]. Qualitative studies involving non-SCAD cardiac-event survivors have also highlighted the importance of loss and grief [32, 33], changes in identity [34], and worries about getting back to one’s previous sense of self [35]. The loss and grief that occurs in chronic illness has been documented since the 1990s [36]. Illness-related losses have been shown to fall into eight distinct categories, namely: loss of bodily function, loss of roles, loss of relationships, loss of activities, loss of an autonomous life, loss of identity, loss of a life imagined, and loss of uplifting emotions [36]. All these losses were articulated by participants in our study. It is important that health professionals acknowledge the losses associated with chronic illness to optimise coping and recovery [36].

Loss of roles and relationships were also evident in reported impacts on work and family life, further extending our understanding of the social impacts of SCAD. Concerns about work capacity were common, and often led to SCAD survivors reducing work time, retiring from the workforce, or feeling inadequate in their work role. Participants also noted their challenges in maintaining parental and household responsibilities, largely due to physical impacts of the condition, most notably extreme fatigue. Worries about the impacts of their health condition on their family were also common, particularly anxiety and stress placed on partners and children due to the sudden and unpredictable nature of the acute event and the condition, reinforcing the findings of the earlier interview study [22]. Negative impacts on both family and work life have also been reported in qualitative studies of non-SCAD cardiac-event survivors [33].

### Clinical implications

The intense emotional impacts of SCAD could be ameliorated through improved provision of information and coherent collaborative care from the healthcare system. Evidence shows that uncertainty can be lessened through the provision of clear and relevant information as well as by health professionals showing confidence in treating the illness [25, 28]. SCAD survivors need consensus regarding management of SCAD, including the role of medications, and guidelines for resumption of activities. A recent systematic review focusing on SCAD recovery similarly reported a need for guidance on resuming physical activity [17]. The current situation involving inadequate and conflicting information and advice has left SCAD survivors floundering, often leading them to unnecessarily limit their activities, avoid physical exertion and situations which might arouse intense emotions, and abandon prescribed medication regimes. Consistent with previous findings [10], participants commonly turned to the internet in their generally unsuccessful attempts to understand and manage their condition. The present findings also highlight the inadequacy of traditional CR programs for SCAD survivors, pointing to the need for SCAD-tailored CR programs, supporting previous qualitative [22] and quantitative studies [17, 18]. Preliminary evidence suggests that SCAD-tailored CR can improve exercise capacity and psychosocial wellbeing, and provide an avenue for referrals to psychological support [37].

Indeed, the findings of the present study point to a clear and urgent need for provision of psychosocial support for SCAD survivors. This again reiterates the findings of a systematic review that reported high levels of distress in SCAD survivors [17]. SCAD survivors need an opportunity to express their fears and concerns, and to be validated and emotionally supported in their recovery. Given the high re-event rate [3, 38, 39], fear of recurrence is a valid and rational concern, and SCAD survivors need the space to express and explore their anxiety around this issue. While fear of recurrence or death have also been reported amongst non-SCAD cardiac-event survivors [33, 40], it is possible that these concerns are more intense in the face of SCAD’s relatively high recurrence rate. Acceptance and Commitment Therapy (ACT) may be an appropriate approach for assisting survivors in this regard [41]. Psychological counselling could also assist in identifying appropriate protective behaviours and addressing self-imposed and possibly irrational restrictions to their lifestyle, including those regarding physical activity and exercise. Cognitive behaviour therapy (CBT) and meta-cognitive therapy have both been identified as appropriate and effective strategies for supporting non-SCAD cardiac-event survivors in their recovery [42–44] and are likely to be equally relevant for SCAD survivors. A recent pilot study of a CBT-based program for SCAD survivors showed promising results in reducing anxiety and depression [45]. A CBT-based program designed specifically to address uncertainty management and coping in chronic obstructive pulmonary disease patients could also have applicability for SCAD patients [46]. Participants in our study expressed a desire for group support, noting that they would benefit from the experience and wisdom of fellow SCAD survivors, particularly those who are further along in their recovery and can therefore provide guidance, encouragement, and inspiration. Benefits of support groups for people with rare diseases are well documented and include, most notably, the importance of meeting and sharing with others with similar experiences, feeling safe to openly express one’s feelings, learning coping skills, and feeling empowered and hopeful [47].

## Conclusion

The present qualitative study has extended previous quantitative investigations of SCAD survivors by providing a more in-depth understanding of the complex, inter-related and often completely overwhelming emotional impacts of an acute SCAD event. The study highlights lack of information as an over-arching issue which exacerbates all other psychosocial impacts, magnifying SCAD survivors’ emotional distress and self-management challenges. The findings point to the urgent need for a coherent approach to information provision, development and delivery of SCAD-specific CR programs, and provision of psychosocial support programs for SCAD survivors. The findings of the present study will be used to develop a questionnaire for use in a larger survey of Australian SCAD survivors, which will quantify the prevalence of the psychosocial impacts presented here. Future studies could also investigate differences between SCAD and non-SCAD cardiac event survivors to further understand the unique experiences of SCAD survivors.
